# Clinical and Radiological Evaluation of Oral and Maxillofacial Status in Patients Undergoing Antiresorptive Therapy and Its Relationship with MRONJ

**DOI:** 10.3390/biomedicines13123054

**Published:** 2025-12-11

**Authors:** Marcela Wiltgen Jeffman, Valesca Sander Koth, Fernanda Gonçalves Salum, Maria Ivete Rockenbach, Aline Cantarelli Morosolli, Karen Cherubini

**Affiliations:** 1 Post-Graduate Program in Dentistry, School of Health and Life Sciences, Pontifical Catholic University of Rio Grande do Sul, Porto Alegre 90619-900, RS, Brazil; marcela.jeffman@edu.pucrs.br (M.W.J.); valesca.koth@edu.pucrs.br (V.S.K.); fernanda.salum@pucrs.br (F.G.S.); 2 Dental Radiology Department, School of Health and Life Sciences, Pontifical Catholic University of Rio Grande do Sul, Porto Alegre 90619-900, RS, Brazil; ivete.rockenbach@pucrs.br (M.I.R.); aline.morosolli@pucrs.br (A.C.M.)

**Keywords:** osteonecrosis, denosumab, bisphosphonates

## Abstract

**Background/Objectives**: Patients undergoing antiresorptive therapy were evaluated, focusing on clinical and radiological features and their relationship with medication-related osteonecrosis of the jaw (MRONJ). **Methods**: Patients were allocated to two groups: MRONJ (*n* = 27) and non-MRONJ (*n* = 139). Clinical evaluation included decayed, missing, and filled teeth (DMFT), number of teeth, periodontal status, prosthesis use, and preventive/therapeutic measures. Imaging analysis included panoramic radiography (PAN) and cone beam computerized tomography (CBCT) to assess MRONJ-related signs. **Results**: The sample showed high levels of DMFT and prosthesis use. There was a tendency of higher prevalence of deeper periodontal pockets in the MRONJ group, with greater need for oral hygiene reinforcement and chlorhexidine prescription. PAN showed higher frequency of osteolysis, persistent alveolar socket, bone sequestration, mandibular canal involvement, periosteal reaction, and sinus involvement in the MRONJ group. Still on PAN, all cases in both groups exhibited a sclerotic trabecular bone pattern. CBCT showed higher frequency of a sclerotic trabecular bone pattern, mandibular canal involvement, and bone sequestration in the MRONJ group. Composite radiographic index modified (CRIm) was higher in the MRONJ group for PAN and CBCT, with low-grade cases in the non-MRONJ and medium-/high-grade cases in the MRONJ group. A positive correlation was found between CRIm and MRONJ staging. **Conclusions**: This study underscores the role of preventive oral care in patients undergoing antiresorptive therapy, highlighting the need for regular periodontal monitoring. Also emphasized is the importance of integrating clinical and radiographic examinations of these patients. Accordingly, CRIm seems to be a reliable tool for MRONJ staging and monitoring.

## 1. Introduction

Medication-related osteonecrosis of the jaw (MRONJ) was first reported by Marx [1] in 2003 and afterward defined as bone exposure or bone that can be probed in the maxillofacial region through an intraoral or extraoral fistula, persisting for more than eight weeks, in individuals with a history of current or previous antiresorptive therapy, and without bone metastasis in the maxillofacial complex or radiotherapy in the head and neck region [2]. This condition significantly affects quality of life, determining oral discomfort, chronic infections, and bone deterioration, which may lead to psychological and functional issues including trouble with speech and eating [3]. Bisphosphonates and denosumab, which are widely used antiresorptive drugs for osteoporosis and bone metastases, have been related to MRONJ [4].

Several risk factors are associated with MRONJ, including the type of medication, as well as the dose and duration of therapy [5]. Dental infections and invasive dental procedures, such as tooth extraction and dental implants, can increase the risk of MRONJ [6,7]. Implementing prophylactic dental measures can reduce potential entry points for pathogens and lower the risk of infection [8]. Therefore, to minimize MRONJ risk, it is advisable to complete any dental procedures needed before initiating antiresorptive therapy [7]. Additional contributing factors include anemia, diabetes, and poorly fitting prostheses [9]. Addressing all these factors is important for prevention, since the effective management of at-risk patients can significantly reduce MRONJ occurrence and progression [5].

MRONJ necrotic areas often appear larger in radiographic examinations than clinically observed, reinforcing that diagnostic criteria should integrate both clinical and radiological findings, making imaging techniques mandatory for MRONJ assessment [10]. Nevertheless, there is no consensus on the best imaging method for this purpose [11]. Panoramic radiography (PAN) is commonly used because of its lower radiation exposure and lower cost. However, it can underestimate lesion size. Cone beam computerized tomography (CBCT), in turn, provides more detailed visualization of lesion extent and can also enable early detection of MRONJ [12].

Patients undergoing antiresorptive therapy can present with radiographic signs such as bone sclerosis, osteolytic areas, thickened lamina dura, persistent alveolar socket, periapical radiolucency, increased mandibular cortex thickness, and widened periodontal ligament space [13]. These findings can help screen patients at risk or having MRONJ and evaluate disease course, magnitude, and progression [14].

Clinical and radiographic evaluations are essential to guide treatment planning in MRONJ. Accordingly, MRONJ was classified into five stages: at risk (asymptomatic; undergoing antiresorptive therapy); stage 0 (no necrotic exposed bone, nonspecific symptoms, or radiographic changes); stage 1 (exposed bone with no infection/inflammation); stage 2 (exposed bone with infection/inflammation); and stage 3 (exposed bone with infection/inflammation and severe complications, such as bone fracture or extraoral fistula) [2]. Given the relevance of radiological examination in staging and treating MRONJ, Walton et al. [15] created the composite radiographic index (CRI). Their aim was to provide a method to more effectively stage and analyze radiographic changes in MRONJ according to the severity of bone alterations. Yfanti et al. [16], in turn, modified Walton’s CRI, creating CRIm, which classifies and rates eight radiological bone features: osteolytic changes, osteosclerosis, periosteal bone formation, bone sequestration, persistent alveolar socket, and additional findings such as maxillary sinus implication, mandibular canal involvement, and jaw fracture.

Considering the importance of oral health in preventing MRONJ and the need to integrate clinical and radiological findings for its diagnostic accuracy, the present study screened the oral and maxillofacial health status of patients undergoing antiresorptive therapy by using clinical and imaging tools. Clinical evaluation comprised dental and periodontal status, while imaging analysis included PAN and CBCT to identify signs consistent with MRONJ, disease staging, and calculating CRIm. The variables analyzed were compared between individuals with and without MRONJ.

## 2. Materials and Methods

This study was approved by the Research Ethics Committee of Pontifical Catholic University of Rio Grande do Sul (PUCRS), protocol CAAE #77785924.0.0000.5336. The sample consisted of 166 adult individuals from 38 to 96 years of age, males and females, undergoing antiresorptive therapy, who freely agreed to participate in the study. All participants signed an informed consent form, and the procedures were conducted in accordance with the principles of the Declaration of Helsinki. This was a convenience sample, where the inclusion criteria comprised patients actively undergoing antiresorptive therapy, regardless of route of administration, duration of treatment, or comorbidities. Meanwhile, the exclusion criteria referred to those with a history of head and neck radiotherapy or bone metastasis in the maxillofacial region. Patients were allocated to two groups according to the presence or absence of MRONJ: (1) MRONJ group (*n* = 27) and (2) non-MRONJ group (*n* = 139). The diagnosis of MRONJ was established according to the AAOMS criteria [2]. Demographic data including the sex and age of the patients and details of antiresorptive therapy (drug class, duration, and route of administration) were collected. Clinical and imaging examinations were performed as follows. Imaging examinations (PAN and/or CBCT) were requested on the basis of the patients’ clinical needs. Patients without MRONJ were initially screened using PAN, and CBCT was performed when necessary. For patients diagnosed with MRONJ, CBCT was prioritized.

### 2.1. Clinical Evaluation

Dental evaluation determined the decayed, missing, and filled teeth (DMFT) index. Patients were also subjected to assessment of periodontal status based on community periodontal index modified (CPIm) criteria, by using the CPI probe [17]. Periodontal examination comprised the presence of gingival bleeding on probing and the presence and depth of periodontal pockets. The frequency of individuals was classified according to pocket depth into (0) no pocket; (1) 4 to 5 mm; and (2) 6 mm or more. For each tooth, six sites were examined comprising mesial, middle, and distal regions on buccal/labial and lingual/palatal surfaces. For dental and periodontal screening, 41 individuals with implants or total edentulism were excluded from the analysis. Clinical evaluation also determined the use of total or partial removable prosthesis.

### 2.2. Implementing Preventive and/or Therapeutic Measures

In accordance with their individual requirements, patients were prescribed chemical biofilm control (0.12% chlorhexidine mouthwash), received oral hygiene reinforcement (tongue cleaning, teeth brushing, dental flossing, interdental brushing, and single-tooth brushing), and underwent scaling and root planing. Patients were referred to specialized treatments, including dental restorations, prosthetic rehabilitation, and root canal treatment. Those with MRONJ requiring surgical intervention and debridement were also appropriately directed. Three months later, patients underwent another round of periodontal examinations.

### 2.3. Imaging Analysis

The imaging examinations (PAN and CBCT) were analyzed by a calibrated and blinded observer who did not know to which group or patient the images belonged. After an initial interobserver calibration with two senior radiologists, intraobserver calibration was also conducted. This process involved performing two evaluations of 30 panoramic radiographs and 5 CBCT scans at two different moments, with a seven-day interval between them. Agreement of the two evaluations was tested by intraclass correlation coefficient (r = 0.9) and Kappa coefficient (k = 0.9). All panoramic radiographs and CBCT scans were analyzed using the 3D Slicer 5.7.0 software (The Slicer Community, Boston, MA, USA).

**Figure 1:**
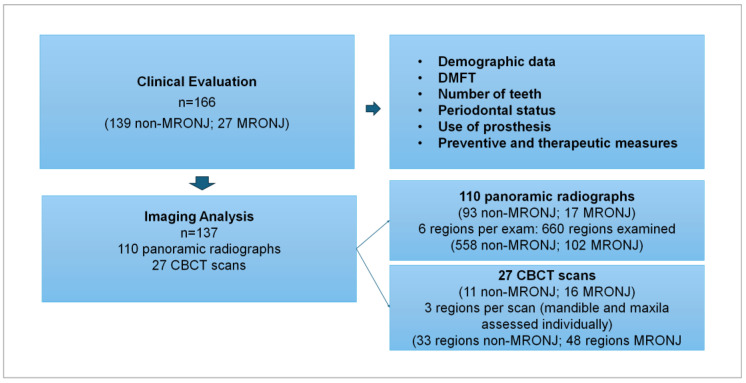
Flowchart illustrating the sample analyzed in the clinical evaluation and imaging analysis. CBCT = cone beam computerized tomography; DMFT = decayed, missing, and filled teeth; MRONJ = medication-related osteonecrosis of the jaw.

A total of 110 panoramic radiographs and 27 CBCT scans were available for analysis (Figure 1). These examinations were evaluated under standardized conditions, with room lights off, always using the same computer, software, and sequence of examination. Three analyses were conducted: (1) the presence of bone features consistent with antiresorptive therapy/MRONJ; (2) the application of CRIm to determine the severity of the changes; and (3) disease staging by adopting the American Association of Oral and Maxillofacial Surgeons (AAOMS) criteria [2].

**Figure 2:**
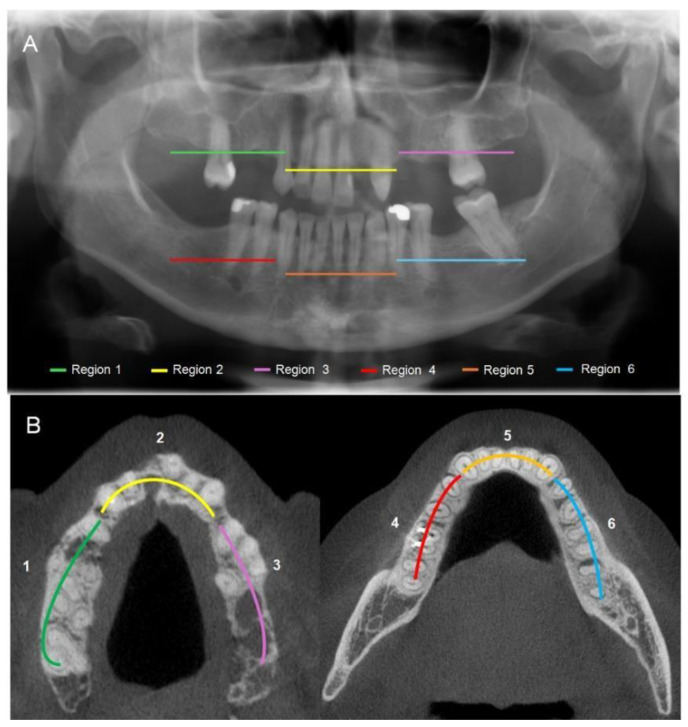
Analysis of panoramic radiography (**A**) and cone beam computerized tomography (**B**): the mandible and the maxilla were divided into six regions (maxilla: right, anterior, and left regions; mandible: right, anterior, and left regions).

PAN and CBCT were analyzed by dividing each jaw into regions: the maxilla and mandible were each divided into right, anterior, and left regions (Figure 2). The anterior regions were defined as the areas between the canines. The posterior maxilla and mandible included the regions extending beyond the canines, covering the premolars and molars, and reaching the maxillary tuberosity and mandibular ramus, respectively. The analysis encompassed the entire visible bone structure of both the maxilla and mandible. In CBCT analysis, the mandible and maxilla were evaluated separately, comprising 3 anatomic regions per scan.

Osteolysis, osteosclerosis, periosteal reaction, bone sequestration, persistent alveolar socket, and “other findings” (pathological fracture, sinus implication, and mandibular canal involvement) were scored as “0” for absent; “1” for localized: lesion extending up to 1 cm; and “2” for extensive: lesions larger than 1 cm or in cases of multiple lesions [16]. CRIm was determined by summing up the individual scores for each radiological feature for each jaw and region examined, yielding a theoretical range of 0–12. The variables pathological fracture, sinus implication, and mandibular canal involvement were grouped under “other findings” and treated as a single feature in the formula calculation [16]. The frequency of the radiographic findings, categorized as present or absent, was evaluated including three additional findings: sclerotic trabecular bone pattern, lamina dura thickening, and cortical bone perforation [18]. The observer was provided with a specialized answer sheet for each panoramic radiograph and CBCT scan, where the location (jaw; region) and radiologic features and the final CRIm score were recorded. In CBCT analysis, each jaw was evaluated in sagittal, axial, and coronal sections. The cases were classified according to CRIm severity: low severity ranges from 0 to 3, medium severity from 4 to 8, and high severity from 9 to 12 [16]. The sample was classified into MRONJ stages: (1) at risk; (2) stage 0; (3) stage 1; (4) stage 2; and (5) stage 3 [2].

### 2.4. Statistical Analysis

Data analysis included both descriptive statistics (frequency, mean, standard deviation, graphs, and tables) and inferential statistics. The Shapiro–Wilk test was applied to assess data distribution. For non-normally distributed data, comparisons between MRONJ and non-MRONJ groups were performed using the Mann–Whitney test. Categorical variables were compared using the chi-square test or Fisher’s exact test. Analysis comparing baseline and three-month clinical examination was performed using the Wilcoxon and McNemar tests. Correlations were assessed using Spearman’s or Pearson’s coefficient. All analyses were conducted in SPSS 21.0 (Statistical Package for the Social Sciences, IBM Co., Armonk, NY, USA) with a significance level of 5%. Graphs were generated using GraphPad Prism 5 (GraphPad Software, San Diego, CA, USA).

## 3. Results

### 3.1. Demographic Data

**Figure 3:**
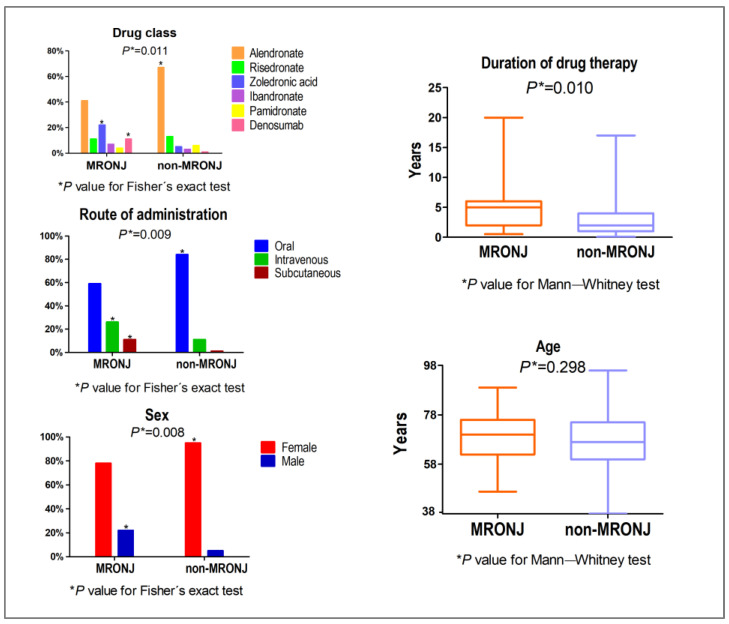
Distribution of the sample according to sex and age of the patients, drug class, route of administration, and duration of therapy in the MRONJ and non-MRONJ groups. Results are considered significant if *p* ≤ 0.05.

In both groups, most of the patients were female. However, the non-MRONJ group had a higher frequency of females than the MRONJ group, which had a higher frequency of males compared to the non-MRONJ group (*p* = 0.008, Fisher’s exact test). Age distribution did not significantly differ between the groups (*p* = 0.298, Mann–Whitney test). Compared to non-MRONJ, the MRONJ group exhibited a longer duration of treatment (*p* = 0.01, Mann–Whitney test) and a higher prevalence of zoledronic acid and denosumab use, while the non-MRONJ group had a higher prevalence of patients taking alendronate (*p* = 0.011, Fisher’s exact test). These results are presented in Figure 3.

### 3.2. Clinical Analysis

#### 3.2.1. Dental Status

**Figure 4:**
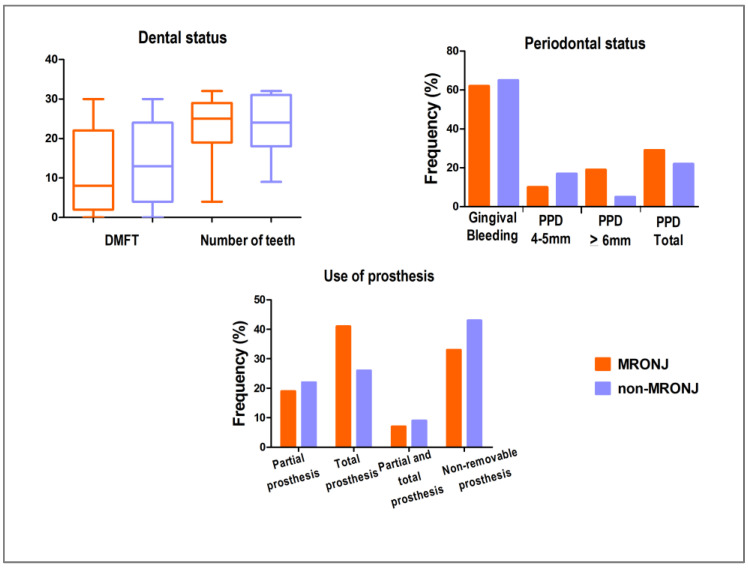
Dental status, periodontal status, and use of prosthesis in the MRONJ and non-MRONJ groups. There was no significant difference between the groups for DMFT index and number of teeth (Mann–Whitney test, *p* > 0.05), gingival bleeding, and total periodontal pocket depth (PPD) (chi-square, *p* > 0.05). A trend was observed toward a higher prevalence of PPD 4–5 mm in the non-MRONJ group, and PPD ≥ 6 mm in the MRONJ group (chi-square test, *p* = 0.059). The frequency of total and partial prosthesis use did not differ significantly between the groups (chi-square, *p* > 0.05). DMFT = decayed, missing, and filled teeth.

The DMFT index (*p* = 0.528) and the number of teeth (*p* = 0.284) did not significantly differ between the MRONJ and non-MRONJ groups (Mann–Whitney, α = 0.05). In the periodontal examination, the frequency of gingival bleeding did not significantly differ between the groups (*p* = 0.761). However, a periodontal pocket depth (PPD) of 4–5 mm was more prevalent in the non-MRONJ, while a PPD ≥ 6 mm was more prevalent in the MRONJ group (*p* = 0.059, chi-square test). The frequency of total and partial prosthesis use did not significantly differ between the groups (*p* = 0.479, chi-square test). These results are presented in Figure 4.

#### 3.2.2. Therapeutic and Preventive Measures

**Figure 5:**
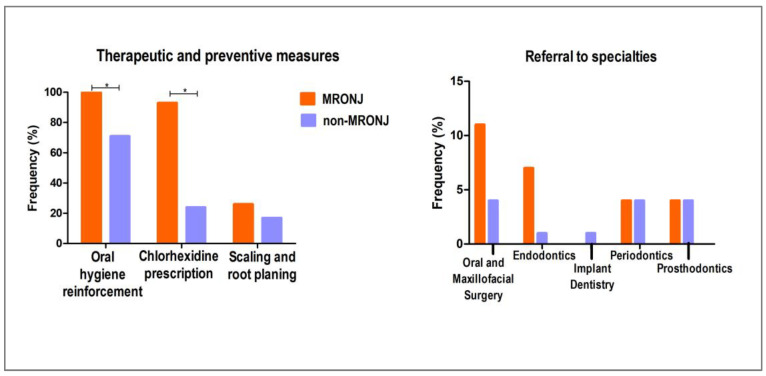
Therapeutic/preventive measures and referral to specialties in the MRONJ and non-MRONJ groups. The need for oral hygiene reinforcement and chlorhexidine prescription was higher in the MRONJ group (Fisher’s exact test, * *p* < 0.05). Scaling and referral to specialties did not significantly differ between the groups (Fisher’s exact test, *p* > 0.05).

The MRONJ group had more patients requiring oral hygiene reinforcement and chlorhexidine prescription compared to the non-MRONJ group (*p* < 0.05). Scaling and root planing was slightly more frequent in the MRONJ group, but the difference was not significant. Also, there were no significant differences in referrals to specialties (*p* > 0.05, Fisher’s exact test; Figure 5).

#### 3.2.3. Clinical Re-Evaluation After Implementing Preventive and Therapeutic Measures

**Figure 6:**
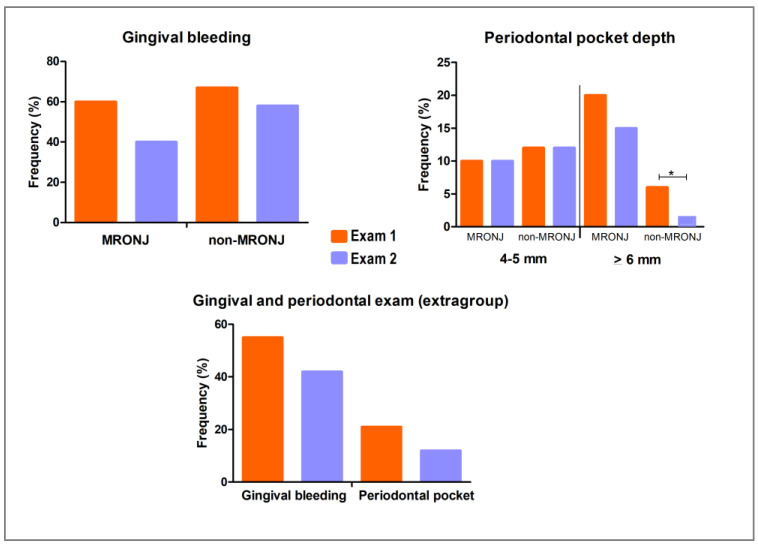
Periodontal status before and after implementing preventive and therapeutic measures in the MRONJ and non-MRONJ groups, based on intragroup and overall sample analyses. Gingival bleeding did not differ significantly between baseline and three-month follow-up (McNemar test, *p* > 0.05). The proportion of periodontal pockets ≥6 mm decreased significantly at the three-month follow-up (Wilcoxon test, * *p* < 0.05), while no significant difference was observed for pockets measuring 4–5 mm (Wilcoxon test, *p* > 0.05).

No significant difference was observed in gingival bleeding when comparing baseline and three-month clinical evaluations either in intragroup or overall sample analyses (*p* > 0.05, McNemar test; Figure 6). On the other hand, the frequency of ≥6 mm periodontal pocket depth decreased significantly in the non-MRONJ group (*p* = 0.046), while no significant differences were observed in the MRONJ group (*p* = 0.577, Wilcoxon test). Thirty-three individuals did not attend the second examination and were excluded from this analysis.

### 3.3. Imaging Analysis

#### 3.3.1. Bone Features on Panoramic Radiography (PAN)

On PAN analysis, osteolysis (*p* = 0.000) and persistent alveolar socket (*p* = 0.004) were significantly more frequent in the MRONJ group than in the non-MRONJ, and bone sequestration, mandibular canal involvement, periosteal reaction, and sinus implication were observed only in the MRONJ group (*p* < 0.05). Osteosclerosis and thickening of the lamina dura did not differ between the groups (*p* > 0.05, Fisher’s exact test; α = 0.05). All cases showed sclerotic trabecular bone pattern, whereas there were no cases of cortical bone perforation and pathological fracture (Table 1).

#### 3.3.2. Bone Features on Cone Beam Computerized Tomography (CBCT)

On CBCT, sclerotic trabecular bone pattern (*p* = 0.003), mandibular canal involvement (*p* = 0.009), and bone sequestration (*p* = 0.009) were significantly more frequent in the MRONJ group. The frequency of osteosclerosis, osteolysis, lamina dura thickening, cortical perforation, periosteal reaction, persistent alveolar socket, and sinus implication did not differ between the groups (*p* > 0.05, Fisher’s exact test). No patient had pathological fractures (Table 2).

#### 3.3.3. Composite Radiographic Index Modified (CRIm)

##### Panoramic Radiography (PAN) and Cone Beam Computerized Tomography (CBCT)

**Figure 7:**
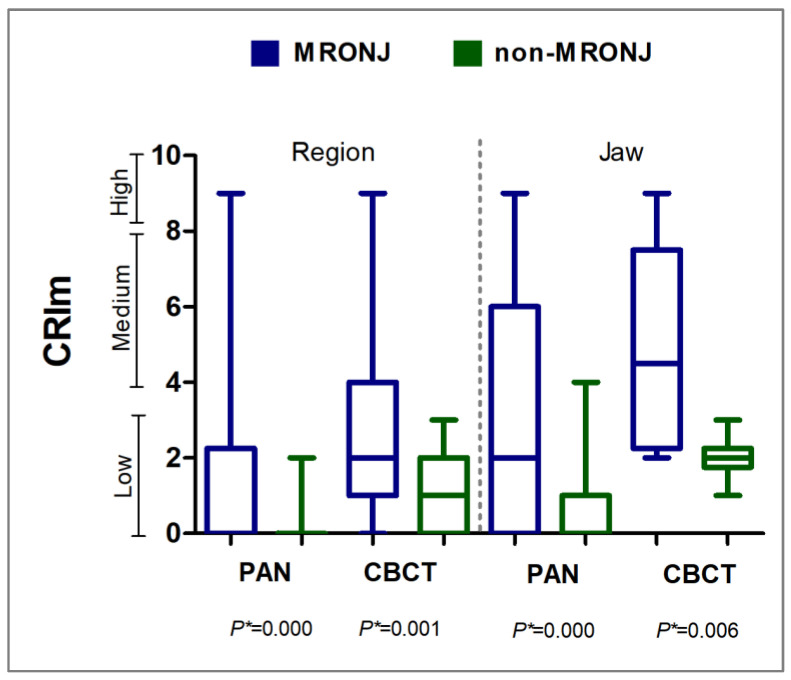
Composite radiographic index modified (CRIm) by region and by jaw on panoramic radiography (PAN) and cone beam computerized tomography (CBCT) in the MRONJ and non-MRONJ groups. * *p* value for Mann–Whitney test; α = 0.05.

**Figure 8:**
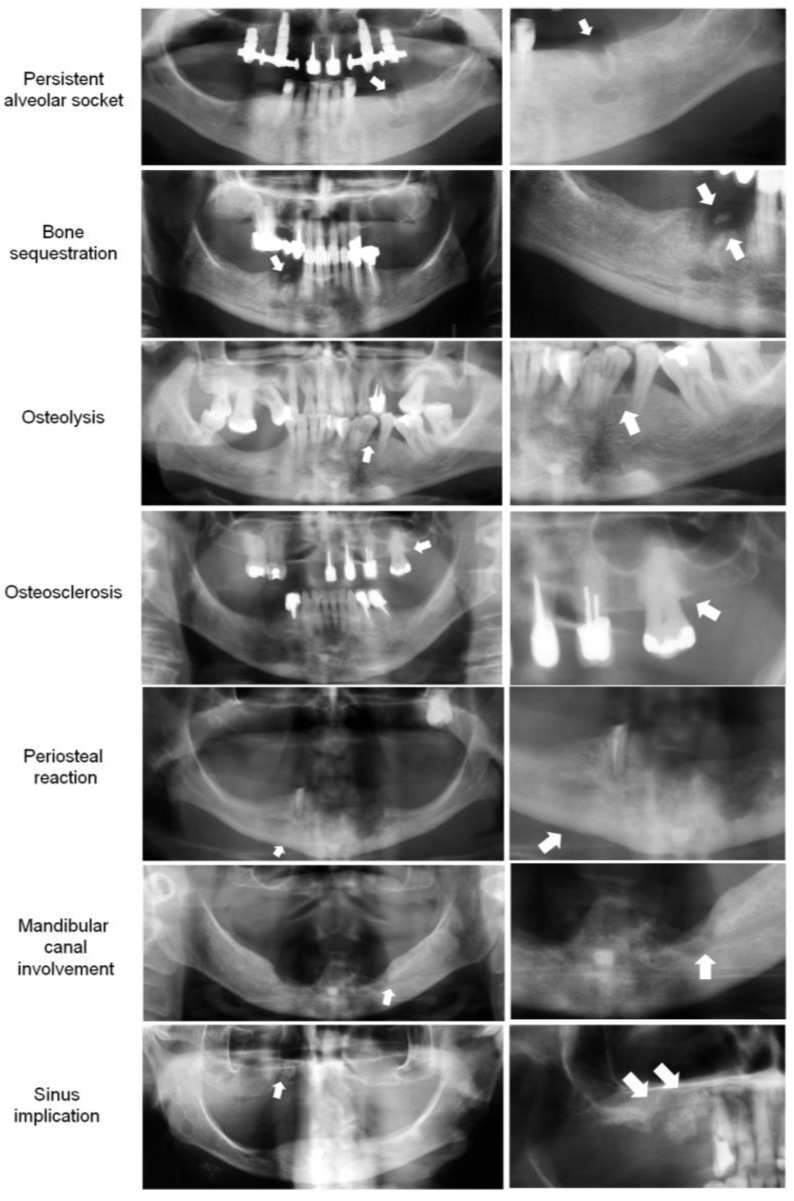
Radiographic signs in the composite radiographic index modified (CRIm) analysis. The right column shows a close-up view of the feature represented in the respective image of panoramic radiography in the left column.

**Figure 9:**
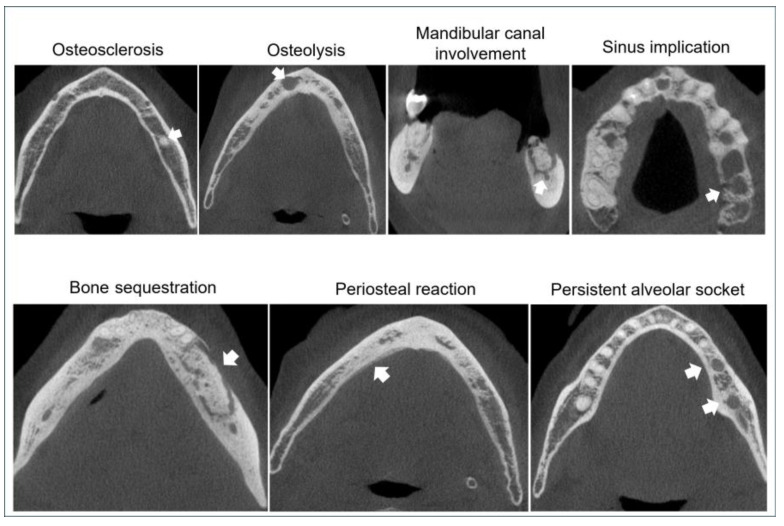
Bone features in the composite radiographic index modified (CRIm) analysis on cone beam computerized tomography (CBCT).

The MRONJ group showed a higher CRIm compared to the non-MRONJ group in both PAN and CBCT either for jaws or regions (*p* < 0.05, Mann–Whitney; Figure 7). Figure 8 and Figure 9 illustrate the bone features analyzed. Table 3 depicts the sample distribution according to the CRIm grades. On the PAN and CBCT analysis, either by region or by jaw, the MRONJ group had low-, medium-, and high-grade CRIm cases, while the non-MRONJ group had mostly low-grade cases with only one medium-grade on PAN (*p* < 0.05).

#### 3.3.4. CRIm Distribution According to Anatomical Region

**Figure 10:**
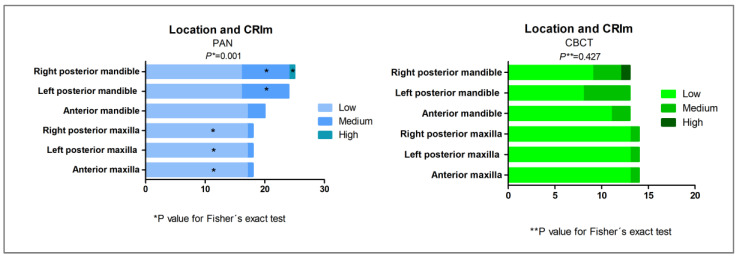
Distribution of the sample according to the composite radiographic index modified (CRIm) severity (low, medium, and high), considering the anatomical regions assessed on panoramic radiography (PAN) and cone beam computerized tomography (CBCT). Statistically significant differences are indicated by (*) for Fisher’s exact test and adjusted residual analysis.

PAN revealed significant differences in CRIm distribution between the anatomical regions, with higher CRIm scores observed in the right posterior mandible and lower CRIm scores in the maxilla (*p* = 0.001). On CBCT, the distribution of CRIm scores across the anatomical regions did not show a significant difference (*p* = 0.427, Fisher’s exact test; Figure 10).

#### 3.3.5. AAOMS Staging

There was a positive correlation between CRIm and MRONJ staging in both PAN (*r* = 0.743) and CBCT (*r* = 0.877) analyses. Table 4 depicts the distribution of the sample according to the MRONJ stages, where most cases in stage zero were classified into low or medium CRIm; most in stages 1 and 2 were in medium CRIm, while in stage 3, the cases were distributed into medium and high CRIm.

## 4. Discussion

The literature emphasizes the importance of combining clinical assessment with structured radiographic analysis to better diagnose MRONJ, especially in early stages [2,11]. While radiographic features such as trabecular sclerosis, bone sequestration, cortical disruption, mandibular canal involvement, and persistent alveolar socket have been repeatedly associated with MRONJ, most studies have focused on descriptive interpretation rather than proposing standardized or quantitative tools for staging or risk stratification [12,16]. In this context, analyzing panoramic and CBCT findings alongside clinical indicators may contribute to a more comprehensive appraisal of bone patterns in antiresorptive users. Therefore, this study offers an integrated view of clinical status and imaging alterations and explores the potential of applying a structured radiographic index (CRIm) to systematize bone changes associated with antiresorptive therapy, including MRONJ severity and disease monitoring.

Dental status, including DMFT, number of teeth, and use of prostheses, did not significantly differ between the MRONJ and non-MRONJ groups. In both groups, most patients exhibited high levels of dental caries and tooth loss. This scenario is likely related to the age of the patients, who belong to a population with a significant history of dental decay and tooth loss early in life, regardless of current medication use and comorbidities [19]. Considering periodontal status, the MRONJ group showed a tendency for a higher prevalence of deeper periodontal pockets. This finding reinforces the idea of periodontal disease being an important risk factor for MRONJ that must be addressed as a preventive measure in patients undergoing antiresorptive therapy [20]. The result is corroborated by the greater need for therapeutic and preventive measures in the MRONJ group, where patients required more frequent prescriptions for chemical biofilm control and reinforcement of oral hygiene. Nevertheless, this could also be due to the presence and management of MRONJ itself [21]. Still, the frequency of referral to specialties did not differ between the groups, which seems to disagree with the worse periodontal condition of the MRONJ group. Nevertheless, it is important to consider here that a great part of the sample was not referred to the periodontist because they received periodontal treatment provided by our team.

The clinical evaluation at baseline and the three-month follow-up did not significantly differ in terms of the prevalence of gingival bleeding, either in intragroup or extragroup analysis. Additionally, periodontal pocket depth prevalence did not change in the MRONJ group between baseline and three-month follow-up, whereas the prevalence of ≥6 mm periodontal pockets significantly decreased in the non-MRONJ group. This result suggests some demotivation among patients in the MRONJ group, possibly due to oral hygiene limitations caused either by MRONJ lesions or the comorbidities and systemic conditions of the patients in this group [22], as many of them were taking zoledronic acid and denosumab for a long period of time. Alternatively, it could be related to the short interval between the two evaluations. Maybe a third examination conducted after a longer period of follow-up could have shown better improvement in the periodontal picture. However, despite the lack of significance, there was a slight reduction in gingival bleeding in the MRONJ group. This finding and the decreased frequency of ≥6 mm pocket in the non-MRONJ group indicate that the preventive and therapeutic measures implemented during the study had a positive effect on periodontal health, though further intervention may be needed to significantly improve periodontal outcomes in both groups.

On PAN analysis, all cases exhibited a sclerotic trabecular bone pattern, while on CBCT, this feature was more prevalent in the MRONJ group. This bone feature is described in patients undergoing antiresorptive treatment regardless of MRONJ occurrence, which explains the high prevalence in both groups [23]. This finding aligns with previous studies that report the correlation between bone density and imbalance in bone remodeling during antiresorptive therapy [24]. However, the discrepancy between PAN and CBCT could be related to the two-dimensional image provided by PAN, which may have given rise to the perception of sclerosis across all cases because of overlapping of anatomical structures [11]. In contrast, CBCT offers a three-dimensional assessment, allowing for a more detailed evaluation of bone architecture [25].

Bone sequestration, sinus implication, periosteal reaction, and mandibular canal involvement occurred exclusively in the MRONJ group on both PAN and CBCT, aligning with established MRONJ imaging characteristics [2]. Bone sequestration and mandibular canal involvement results were statistically significant for both examinations; however, sinus implication and periosteal reaction results were significant only on PAN. Similarly, osteolysis and persistent alveolar socket were significantly more prevalent only on PAN, not on CBCT. This disagreement can be attributed to the higher spatial resolution of this modality, which minimizes false negatives by capturing subtle structural changes less apparent on PAN [12,26], or even to CBCT’s smaller sample size in our study. Moreover, even though CBCT is considered the gold-standard imaging technique for evaluating MRONJ [12], it is plausible that it has limitations that can interfere with the results. One significant limitation, for instance, is the artifacts, especially those caused by metallic restorations and dental implants, which sometimes allow PAN to perform better than CBCT [27].

Cortical bone perforation was detected exclusively on CBCT, with no significant differences observed between the groups. This suggests that PAN has limitations in detecting cortical changes [28], reinforcing the higher accuracy of tomography [29]. Lamina dura thickening and osteosclerosis showed no intergroup differences in imaging modalities. The intensity of lamina dura thickening has been suggested as an indicator of MRONJ presence or imminence [30]. Maybe if we had taken such measures, the results for this variable could have shown a difference between the groups. Also, the uniform distribution of both lamina dura thickening and osteosclerosis may indicate a limited observation period for detecting significant differences [18], or once again, the lack of significance can be attributed to the association of these imaging features with antiresorptive use independent of MRONJ occurrence. Eventually, no pathological fractures were identified in the sample, which was in agreement with clinical MRONJ staging.

The higher CRIm observed in the MRONJ group compared to the non-MRONJ group and its positive correlation to AAOMS MRONJ staging support CRIm as a reliable index for MRONJ, not only for CBCT but also for PAN changes. CRIm holds significant clinical and research relevance in the context of staging and monitoring MRONJ, giving a quantitative analysis of bone lesions [16]. Future studies investigating the correlation of CRIm with bone metabolism markers such as C-terminal cross-linked telopeptide (CTX), alkaline phosphatase, and osteocalcin would be of help in checking the performance of CRIm in MRONJ evaluation.

It is important to recall that this study was conducted in a real-world clinical setting, which may have introduced heterogeneity and limited control over some clinical variables. The number of MRONJ cases was relatively small compared to the total sample—although consistent with the low prevalence of the condition—restricting the possibility of more advanced statistical modeling and broader validation of radiographic predictors. These aspects are intrinsic to the design and nature of the study and reinforce the need for future prospective and multicenter investigations to refine and validate the proposed approach.

Nevertheless, regardless of its limitations, this study underscores the critical role of preventive oral healthcare in patients undergoing antiresorptive therapy and reinforces the value of regular periodontal monitoring as an essential component of comprehensive risk assessment. It highlights the importance of integrating clinical findings with structured radiographic interpretation to enhance the understanding, staging, and monitoring of MRONJ. Within this context, CRIm emerges as a promising complementary tool. Further studies are needed to better evaluate and validate this index as capable of systematizing radiographic indicators into a quantitative framework that may support longitudinal follow-up and strengthen radiographic reasoning.

Eventually, by promoting a preventive, imaging-informed, and multidisciplinary approach, the integrative model of our study contributes to the advancement of MRONJ evaluation and may guide the refinement of radiographic-based risk stratification, staging strategies, and patient management protocols. Overall, the alignment of clinical and radiographic perspectives presented in this study provides valuable insights for early recognition, structured disease assessment, and future development of imaging-based tools to support preventive, diagnostic, and monitoring purposes in patients at risk for MRONJ.

## Figures and Tables

**Table 1:** Frequency of bone features on panoramic radiography in the MRONJ and non-MRONJ groups.

MRONJ			Non-MRONJ		Total		
Feature	(*n* = 102)		(*n* = 558)		(*n* = 660)		*p* *
	*n*	%	*n*	%	*n*	%	
Sclerotic trabecular bone pattern	102	100	558	100	660	100	-
Lamina dura thickening	42	42	202	36	244	37	0.372
Osteolysis	37	36	47	8	84	13	0.000
Bone sequestration	16	16	0	0	16	2	0.000
Mandibular canal involvement	15	15	0	0	15	2	0.000
Osteosclerosis	13	13	53	10	66	10	0.368
Periosteal reaction	11	11	0	0	11	2	0.000
Persistent alveolar socket	9	9	14	3	23	4	0.004
Sinus implication	2	2	0	0	2	1	0.024
Cortical bone perforation	0	0	0	0	0	0	-
Pathological fracture	0	0	0	0	0	0	-

* *p* value for Fisher’s exact test, α = 0.05; “*n*” corresponds to the number of jaw regions analyzed; in the MRONJ group, 17 panoramic radiographs were analyzed; in the non-MRONJ group, 93 panoramic radiographs were analyzed.

**Table 2:** Frequency of bone features on cone beam computerized tomography (CBCT) in MRONJ and non-MRONJ groups.

	MRONJ		Non-MRONJ		Total		
Feature	(*n* = 48)		(*n* = 33)		(*n* = 81)		*p* *
	*n*	%	*n*	%	*n*	%	
Sclerotic trabecular bone pattern	48	100	27	82	75	93	0.003
Osteosclerosis	37	77	19	58	56	69	0.087
Osteolysis	31	65	14	42	45	56	0.069
Lamina dura thickening	29	60	17	52	46	57	0.497
Cortical bone perforation	10	21	2	6	12	15	0.110
Mandibular canal involvement	9	19	0	0	9	11	0.009
Bone sequestration	9	19	0	0	9	11	0.009
Periosteal reaction	5	10	0	0	5	6	0.076
Persistent alveolar socket	5	10	2	6	7	9	0.695
Sinus implication	3	6	0	0	3	4	0.267
Pathological fracture	0	0	0	0	0	0	-

*p value for Fisher’s exact test, α = 0.05. “*n*” corresponds to the number of jaw regions analyzed; in the MRONJ group, 16 CBCT scans were analyzed; in the non-MRONJ group, 11 CBCT scans were analyzed.

**Table 3:** Distribution of MRONJ and non-MRONJ samples according to CRIm on panoramic radiography (PAN) and cone beam computerized tomography (CBCT), considering regions and jaws.

			Region							Jaw				
CRIm	MRONJ		Non-MRONJ		Total		*p* *	MRONJ		Non-MRONJ		Total		*p* *
	*n*	%	*n*	%	*n*	%		*n*	%	*n*	%	*n*	%	
PAN														
Low	79	76	**558**	100	637	96		19	56	**185**	99	204	92	
Medium	**22**	22	0	0	22	3	0.003	**14**	41	1	1	15	7	0.000
High	**1**	2	0	0	1	1		**1**	3	0	0	1	1	
Total	102	100	558	100	660	100		34	100	186	100	220	100	
CBCT														
Low	34	71	**33**	100	67	83		6	38	**11**	100	17	63	
Medium	**13**	27	0	0	13	16	0.003	**8**	50	0	0	8	30	0.004
High	1	2	0	0	1	1		**2**	12	0	0	2	7	
Total	48	100	33	100	81	100		16	100	11	100	27	100	

MRONJ = medication-related osteonecrosis of the jaws; CRIm = composite radiographic index modified; “*n*” corresponds to the number of regions or jaws analyzed. * *p* value for Fisher’s exact test. Bold values show significance (adjusted residual analysis, α = 0.05).

**Table 4:** Correlation between AAOMS MRONJ staging and composite radiographic index modified (CRIm) in panoramic radiography (PAN) and cone beam computerized tomography (CBCT).

MRONJ Staging (AAOMS)												
	0		1		2		3		Total			
CRIm PAN	*n*	%	*n*	%	*n*	%	*n*	%	*n*	%	*p*	*r*
Low	**88**	95	1	14	2	25	0	0	91	83	0.000 *	0.743 **
Medium	5	5	**4**	57	**5**	63	**1**	50	15	14		
High	0	0	**2**	29	**1**	12	**1**	50	4	3		
Total	93	100	7	100	8	100	2	100	110	100		
CRIm CBCT												
Low	**10**	100	1	33	1	25	0	0	12	60	0.000 *	0.877 ***
Medium	0	0	**2**	67	0	0	0	0	2	10		
High	0	0	0	0	**3**	75	**3**	100	6	30		
Total	10	100	3	100	4	100	3	100	20	100		

* *p* value for Fisher’s exact test. ** *r* value for Spearman’s correlation coefficient, *** *r* value for Pearson’s correlation coefficient. Bold values show significance (adjusted residual analysis, α = 0.05). AAOMS = American Association of Oral and Maxillofacial Surgeons.

## Data Availability

The original contributions presented in this study are included in the article. Further inquiries can be directed to the corresponding author.

## Abbreviations

The following abbreviations are used in this manuscript:

AAOMS	American Association of Oral and Maxillofacial Surgeons
CBCT	cone beam computerized tomography
CPI	community periodontal index
CPIm	community periodontal index modified
CRI	composite radiographic index
CRIm	composite radiographic index modified
CTX	C-terminal cross-linked telopeptide
DMFT	decayed, missing, and filled teeth
MRONJ	medication-related osteonecrosis of the jaw
